# Real-world NUDT15 genotyping and thiopurine treatment optimization in inflammatory bowel disease: a multicenter study

**DOI:** 10.1007/s00535-024-02099-7

**Published:** 2024-04-08

**Authors:** Motoki Makuuchi, Yoichi Kakuta, Junji Umeno, Toshimitsu Fujii, Tetsuya Takagawa, Takashi Ibuka, Miki Miura, Yu Sasaki, Sakuma Takahashi, Hiroshi Nakase, Hiroki Kiyohara, Keiichi Tominaga, Yosuke Shimodaira, Sakiko Hiraoka, Nobuhiro Ueno, Shunichi Yanai, Takeo Yoshihara, Kazuki Kakimoto, Katsuyoshi Matsuoka, Ryohei Hayashi, Sohachi Nanjo, Itaru Iwama, Yoh Ishiguro, Hirofumi Chiba, Katsuya Endo, Takashi Kagaya, Tomohiro Fukuda, Yasuhisa Sakata, Takahiro Kudo, Tomohisa Takagi, Kenichi Takahashi, Makoto Naganuma, Masaru Shinozaki, Noriyuki Ogata, Hiroki Tanaka, Kazuyuki Narimatsu, Haruka Miyazaki, Takashi Ishige, Motoyuki Onodera, Yu Hashimoto, Hiroshi Nagai, Yusuke Shimoyama, Takeo Naito, Rintaro Moroi, Hisashi Shiga, Post-MENDEL study group, Yoshitaka Kinouchi, Akira Andoh, Tadakazu Hisamatsu, Atsushi Masamune

**Affiliations:** 1 Division of Gastroenterology, Tohoku University Graduate School of Medicine, 1-1 Seiryo, Aoba, Sendai, 980-8574 Japan; 2 Department of Medicine and Clinical Science, Graduate School of Medical Sciences, Kyushu University, Fukuoka, Japan; 3 Department of Gastroenterology and Hepatology, Tokyo Medical and Dental University, Tokyo, Japan; 4 Center for Clinical Research and Education/Center for Inflammatory Bowel Disease, Hyogo Medical University, Nishinomiya, Japan; 5 Department of Gastroenterology, Gifu University Graduate School of Medicine, Gifu, Japan; 6 Department of Gastroenterology and Hepatology, Kyorin University School of Medicine, Tokyo, Japan; 7 Department of Gastroenterology, Faculty of Medicine, Yamagata University, Yamagata, Japan; 8 Department of Gastroenterology, Kagawa Prefectural Central Hospital, Takamatsu, Japan; 9 Department of Gastroenterology and Hepatology, Sapporo Medical University School of Medicine, Sapporo, Japan; 10 Division of Gastroenterology and Hepatology, Department of Internal Medicine, Keio University School of Medicine, Tokyo, Japan; 11 Department of Gastroenterology, Dokkyo Medical University, Tochigi, Japan; 12 Department of Gastroenterology and Neurology, Akita University, Akita, Japan; 13 Department of Gastroenterology and Hepatology, Okayama University Graduate School of Medicine, Dentistry and Pharmaceutical Sciences, Okayama, Japan; 14 Division of General Medicine, Asahikawa Medical University Hospital, Asahikawa, Japan; 15 Division of Gastroenterology and Hepatology, Department of Internal Medicine, Iwate Medical University, Morioka, Japan; 16 Department of Gastroenterology and Hepatology, Osaka University Graduate School of Medicine, Osaka, Japan; 17 Second Department of Internal Medicine, Osaka Medical and Pharmaceutical University, Osaka, Japan; 18 Division of Gastroenterology and Hepatology, Department of Internal Medicine, Toho University Sakura Medical Center, Chiba, Japan; 19 Department of Gastroenterology, Hiroshima University Hospital, Hiroshima, Japan; 20 Third Department of Internal Medicine, Graduate School of Medicine, University of Toyama, Toyama, Japan; 21 Division of Gastroenterology and Hepatology, Saitama Children’s Medical Center, Saitama, Japan; 22 Division of Clinical Research, Hirosaki General Medical Center, NHO, Hirosaki, Japan; 23 Department of Gastroenterology, Iwate Prefectural Isawa Hospital, Oshu, Japan; 24 Division of Gastroenterology, Tohoku Medical and Pharmaceutical University School of Medicine, Sendai, Japan; 25 Department of Gastroenterology, NHO Kanazawa Medical Center, Kanazawa, Japan; 26 Center for Advanced IBD Research and Treatment, Kitasato University Kitasato Institute Hospital, Tokyo, Japan; 27 Division of Gastroenterology, Department of Internal Medicine, Faculty of Medicine, Saga University, Saga, Japan; 28 Department of Pediatrics, Juntendo University Faculty of Medicine, Tokyo, Japan; 29 Department of Molecular Gastroenterology and Hepatology, Kyoto Prefectural University of Medicine, Kyoto, Japan; 30 Department of Colorectal Surgery, Tohoku Rosai Hospital, Sendai, Japan; 31 Third Department of Internal Medicine, Kansai Medical University, Hirakata, Japan; 32 Saitama Gastroenterology Clinic, Saitama, Japan; 33 Digestive Disease Center, Showa University Northern Yokohama Hospital, Yokohama, Japan; 34 Sapporo IBD Clinic, Sapporo, Japan; 35 Department of Internal Medicine, National Defense Medical College, Tokorozawa, Japan; 36 Division of Gastroenterology, Department of Internal Medicine, Kobe University Graduate School of Medicine, Kobe, Japan; 37 Department of Pediatrics, Gunma University Graduate School of Medicine, Maebashi, Japan; 38 Osaki Citizen Hospital, Osaki, Japan; 39 Department of Gastroenterology and Hepatology, Gunma University Graduate School of Medicine, Maebashi, Japan; 40 Student Healthcare Center, Institute for Excellence in Higher Education, Tohoku University, Sendai, Japan; 41 Division of Gastroenterology and Hematology, Department of Medicine, Shiga University of Medical Science, Otsu, Japan

**Keywords:** NUDT15, Thiopurine, Azathioprine, 6-mercaptopurine, Adverse event

## Abstract

**Background:**

This study evaluated the effectiveness of NUDT15 codon 139 genotyping in optimizing thiopurine treatment for inflammatory bowel disease (IBD) in Japan, using real-world data, and aimed to establish genotype-based treatment strategies.

**Methods:**

A retrospective analysis of 4628 IBD patients who underwent NUDT15 codon 139 genotyping was conducted. This study assessed the purpose of the genotyping test and subsequent prescriptions following the obtained results. Outcomes were compared between the Genotyping group (thiopurine with genotyping test) and Non-genotyping group (thiopurine without genotyping test). Risk factors for adverse events (AEs) were analyzed by genotype and prior genotyping status.

**Results:**

Genotyping test for medical purposes showed no significant difference in thiopurine induction rates between Arg/Arg and Arg/Cys genotypes, but nine Arg/Cys patients opted out of thiopurine treatment. In the Genotyping group, Arg/Arg patients received higher initial doses than the Non-genotyping group, while Arg/Cys patients received lower ones (median 25 mg/day). Fewer AEs occurred in the Genotyping group because of their lower incidence in Arg/Cys cases. Starting with < 25 mg/day of AZA reduced AEs in Arg/Cys patients, while Arg/Arg patients had better retention rates when maintaining ≥ 75 mg AZA. Nausea and liver injury correlated with thiopurine formulation but not dosage. pH-dependent mesalamine reduced leukopenia risk in mesalamine users.

**Conclusions:**

NUDT15 codon 139 genotyping effectively reduces thiopurine-induced AEs and improves treatment retention rates in IBD patients after genotype-based dose adjustments. This study provides data-driven treatment strategies based on genotype and identifies risk factors for specific AEs, contributing to a refined thiopurine treatment approach.

**Supplementary Information:**

The online version contains supplementary material available at 10.1007/s00535-024-02099-7.

## Introduction

Inflammatory bowel disease (IBD), encompassing Crohn’s disease (CD), and ulcerative colitis (UC) are characterized by relentless chronic inflammation of the intestinal tract. Thiopurine medications, such as azathioprine (AZA) and 6-mercaptopurine (6-MP), have long served as pivotal maintenance therapies for IBD remission [1]. Despite the emergence of novel biologic agents in IBD treatment, thiopurine medications retain their significance owing to their cost-effectiveness and well-established evidence base [2–4].

However, thiopurine therapy is hampered by drug-specific adverse events (AEs), including leukopenia and alopecia. In 2014, a compelling correlation was uncovered between thiopurine-induced leukopenia and the R139C polymorphism of the NUDT15 gene, involving an arginine (Arg) to cysteine (Cys) substitution at position 139 [5–7]. Subsequently, we conducted a retrospective analysis within the framework of the MENDEL study, a multicenter research endeavor in Japan, to explore the connection between NUDT15 polymorphisms and various thiopurine-related AEs [8]. Within this study, among the assorted gene polymorphisms of NUDT15, the codon 139 polymorphism emerged as the most salient predictor of severe leukopenia and profound alopecia [9, 10]. As a result, since February 2019, all Japanese patients commencing thiopurine therapy for the first time have been offered NUDT15 codon 139 genotyping, which is covered by public medical insurance [11].

A prior prospective intervention study conducted in China underscored the utility of NUDT15 genotyping prior to thiopurine induction [12]. Despite the compelling evidence obtained in a research context, it is imperative to ascertain whether pharmacogenomic testing genuinely mitigates AEs in clinical practice. This study, referred to as the “Post-MENDEL” study, is intended to confirm whether the innovative genetic test developed based on the results of the MENDEL study has effectively reduced AEs and improved treatment safety. Another goal is to elucidate the appropriate use of thiopurines based on genetic polymorphism testing outcomes, encompassing unforeseeable AEs, and strategies for their management. This involves leveraging extensive real-world data from Japan, where routine genetic polymorphism testing in clinical practice was first implemented.

## Materials and methods

### Study population and dataset creation

**Fig. 1:**
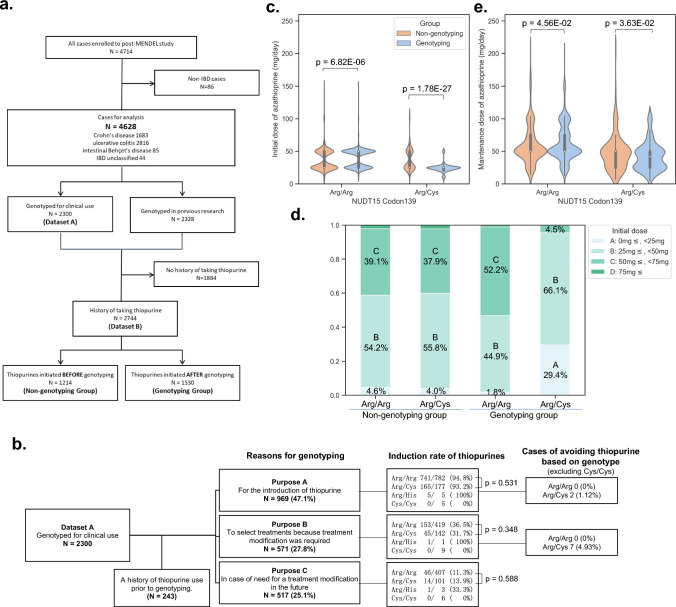
Grouping of subjects and induction status of thiopurines for each group. **a** Study flowchart. Excluding patients with conditions other than inflammatory bowel disease from the enrolled cases, two datasets were created and analyzed according to the purpose of the analysis. **b** Reasons for genotyping and induction rate of thiopurines by genotype. *Excluding Cys/Cys cases, frequency was based on each genotype. **c, d** Violin plots of initial (**c**) and maintenance (**d**) dose of azathioprine stratified by genotype for the Genotyping group (blue) and Non-genotyping group (red). **e** The frequency of the four groups according to the initial dose is shown for each genotype and prior genotyping status. IBD, inflammatory bowel disease

This retrospective study in Japan included individuals diagnosed with IBD who had previously undergone genotyping for NUDT15 codon 139. The study covered the period from July 2020 to January 2022 and involved 39 hospitals. Initially, 4714 patients were enrolled, but 86 were excluded because of medical conditions other than CD, UC, intestinal Behçet’s disease (BD), or IBD, unclassified (IBDU). This led to a final cohort of 4628 patients (Fig. 1a).

In this study, two datasets were created for analysis: Dataset A focused on cases in which NUDT15 genotyping was performed for medical reasons after it became commercially available, exploring the reasons behind the test and how the physician and patient made treatment decisions based on the obtained genotyping results. Dataset B included cases with a history of thiopurine usage, irrespective of prior genotyping test, to assess the clinical course following thiopurine treatment. To evaluate the utility of prior genotyping test, a comparative analysis was conducted in Dataset B. The Genotyping group comprised patients who started thiopurine treatment based on genetic test results, while the Non-genotyping group consisted of patients who initiated thiopurine treatment without a prior genotyping test, but later underwent genotyping primarily for research purposes. This analysis aimed to determine the impact of the genotyping test on treatment decisions and outcomes in individuals with IBD.

This study was approved by the Ethics Committee of Tohoku University School of Medicine (2022-1-027).

### Data collection

Patient information collected for this study included NUDT15 codon 139 genotype, genotyping date, sex, date of birth, and diagnosis. Dataset A, which focused on medical purposes, included data on the purpose of the genetic test, application of thiopurine treatment, and reasons for avoiding treatment based on the genetic test results, if applicable. Dataset B, involving patients with a history of thiopurine use, recorded initial and maintenance thiopurine doses, treatment initiation and discontinuation dates with associated reasons, presence of AEs, AE onset dates, and responses to AEs. Additionally, the use of xanthine oxidase inhibitors and 5-aminosalicylic acid preparations, which affect thiopurine metabolism, was examined, along with specific details on mesalamine formulations and their doses.

### Thiopurine dosing and categorization

Data on the doses and types of thiopurines were collected at the start of treatment, at the time of dose reduction or discontinuation, and at the last observation. The maintenance dose was defined as the longest administered dose for patients treated for at least 26 weeks. Thiopurine doses were calculated by multiplying the 6-MP dose by 2.08 to achieve equivalence with AZA doses, using a molecular weight-based formula [13]. Doses were categorized into four levels: A (very low dose, < 25 mg), B (low dose, 25 mg to < 50 mg), C (standard dose, 50 mg to < 75 mg), and D (high dose, ≥ 75 mg).

### Definitions of adverse events

Here, the term “adverse drug reactions” refers to events that required thiopurine dose reduction or discontinuation, with the attending physician confirming a direct link to thiopurine therapy [8]. The analysis of AE risk factors mainly focused on events within 2 years of starting thiopurine treatment. Leukopenia was defined as Common Terminology Criteria for Adverse Events (CTCAE) Grade 1 or higher (leukocyte count < 3300/mm^3^), and severe leukopenia as Grade 3 or higher (leukocyte count < 2000/mm^3^). Alopecia was categorized as mild (subjective symptoms) or severe (observable by others). Liver injury was defined by elevated liver enzyme levels in blood tests. Pancreatitis was diagnosed through imaging, with separate analysis for hyperamylasemia.

### Genotyping methods

Genotypic data for rs116855232 (R139C) and rs147390019 (R139H) in the NUDT15 gene were employed. All cases genotyped for medical purposes were analyzed using the MEBRIGHT NUDT15 reagent (Medical & Biological Laboratories Co., Ltd., Tokyo, Japan), the only approved diagnostic genotyping test reagent in Japan. Genotyping methods for cases genotyped for research purposes were the TaqMan method, genotyping array, or direct sequencing [8, 10, 14–17].

### Statistical analysis

In Dataset A, we analyzed thiopurine induction rates based on the purpose of the genotyping test and the genotype, focusing on the major genotypes Arg/Arg and Arg/Cys. In Dataset B, we estimated cumulative thiopurine retention rates and the cumulative incidence of thiopurine-related AEs using the Kaplan–Meier method. Comparisons were made between Genotyping and Non-genotyping groups, as well as by genotype and dose. The analysis of Non-genotyping vs. Genotyping groups covered 2 years from thiopurine induction, while long-term maintenance dose analysis spanned 5 years. Genotypes containing the rare allele of histidine (His), namely, Arg/His and Cys/His, were excluded from the statistical calculations due to the limited number of cases.

To address potential confounding effects, subjects were stratified by genotype, and a Cox proportional hazards model was employed. For individual AEs, occurrences of other AEs were considered as observational interruptions. Continuous variables were summarized using median and interquartile range (IQR) and compared using the Kruskal–Wallis test. Mean and standard deviation (SD) were used for specific continuous variables. Categorical variables were summarized by frequency and compared using the *χ*^2^ test. Python 3.11.1, including pandas, lifelines, matplotlib, and seaborn, was used for statistical analyses and data visualization. A significance level of *p* < 0.05 was considered statistically significant.

## Results

### Patient characteristics

A cohort of 4628 individuals was analyzed (Supplementary Table S1). Median age at the time of the genotyping test was 37 years, with 61.8% of the subjects being male. Diagnoses included 2816 (60.8%) with UC, 1683 (36.4%) with CD, 85 with BD, and 44 with IBDU. In Dataset A, comprising 2300 patients genotyped for medical purposes, 77.7% had the genotype of Arg/Arg, 20.7% had Arg/Cys, and 1.2% had Cys/Cys. Dataset B included 2744 patients with a history of thiopurine administration, with 1214 in the Non-genotyping group and 1530 in the Genotyping group (Table 1). No Cys/Cys cases were observed in the Genotyping group. Median starting and maintenance doses were 25 and 50 mg/day, respectively, with lower 5-ASA use in the Genotyping group (*p* = 3.01E−14).

**Table 1:** Patient characteristics of thiopurine users (Dataset B) by prior genotyping

	Non-genotyping	Genotyping	p-Value
Total number of subjects	1214	1530	
Sex, *n* (%)			
Male	769 (63.3)	971 (63.5)	9.80E−01
Female	445 (36.7)	559 (36.5)	
Diagnosis, *n* (%)			
UC	680 (56.0)	973 (63.6)	3.36E−05^*^
CD	507 (41.8)	511 (33.4)	
BD	21 (1.7)	27 (1.8)	
IBDU	6 (0.5)	19 (1.2)	
NUDT15 codon 139, *n* (%)			
Arg/Arg	912 (75.1)	1230 (80.4)	5.63E−14^*^
Arg/Cys	251 (20.7)	291 (19.0)	
Arg/His	2 (0.2)	9 (0.6)	
Cys/His	1 (0.1)	0	
Cys/Cys	48 (4.0)	0	
Age at diagnosis, median (IQR)	26.0 (19.0, 36.0)	27.0 (20.0, 40.0)	7.00E−03^*^
Age at thiopurine induction, median (IQR)	35.0 (26.0, 47.0)	35.0 (23.0, 47.0)	2.11E−01
Body weight (kg), median (IQR)	55.0 (48.0, 64.0)	57.0 (49.0, 65.0)	5.02E−02
Thiopurine drug at induction, *n* (%)			
AZA	1067 (88.0)	1379 (90.2)	7.13E−02
6MP	146 (12.0)	150 (9.8)	
Induction dose (AZA)			
Median (IQR)	25.0 (25.0, 50.0)	25.0 (25.0, 50.0)	6.73E−01
Mean ± SD	36.8 ± 16.6	36.2 ± 14.2	
Concomitant use of 5-ASA, *n* (%)			
Yes	875 (81.8)	1047 (68.6)	3.01E−14^*^
Not used	193 (18.1)	480 (31.4)	
Concomitant use of XO inhibitors, *n* (%)			
Yes	17 (1.4)	20 (1.3)	6.47E−01
Not used	1040 (86.2)	1506 (98.6)	
Thiopurine drug at maintenance, *n* (%)			
6MP	209 (17.5)	192 (12.6)	3.42E−04^*^
AZA	982 (82.5)	1336 (87.4)	
Maintenance dose (AZA)			
Median (IQR)	50.0 (41.6, 62.4)	50.0 (31.2, 62.4)	4.59E−01
Mean ± SD	57.9 ± 30.6	58.5 ± 28.9	

*IQR* interquartile range, *UC* ulcerative colitis, *CD* Crohn’s disease, *BD* intestinal Behçet’s disease, *IBDU* inflammatory bowel disease, unclassified, *AZA* azathioprine, *6MP* 6-mercaptopurine, *5-ASA* 5-aminosalicylic acid, *XO* xanthine oxidase

*p < 0.05

### No difference in thiopurine induction rate between Arg/Arg and Arg/Cys

A total of 243 patients in Dataset A were excluded from the analysis because of a history of thiopurine use prior to genotyping. Among the remaining 2057 patients, 47.1% were tested after deciding to initiate thiopurines (purpose A), 27.8% were tested at the time of selecting treatment because treatment modification was required (purpose B), and 25.1% were tested for future use without immediate plans to change their therapy (purpose C) (Fig. 1b).

Thiopurine induction rates varied among genotyping purposes A–C, with rates of 94.0%, 34.9%, and 11.8%, respectively. No significant differences in thiopurine induction rates were seen between Arg/Arg and Arg/Cys genotypes in any group. Nine patients chose not to start treatment with thiopurines after genotyping; all of these had the Arg/Cys genotype (excluding contraindicated Cys/Cys cases). Reasons for choosing not to start thiopurines included prioritizing other treatments (7 cases), fertility concerns (1 case), older age (1 case), and miscellaneous reasons (1 case).

### Initial doses were increased for Arg/Arg and decreased for Arg/Cys

Initial and maintenance thiopurine doses by genotype were summarized in Fig. 1c, d and Supplementary Table S2. In the Non-genotyping group, the median initial dose was 25 mg/day for all genotypes. In contrast, the Genotyping group had a median initial dose of 50 mg/day for Arg/Arg and 25 mg/day for Arg/Cys, which differed significantly (*p* = 3.68E−68). Comparing doses between groups in each genotype, Arg/Arg patients in the Genotyping group started at higher doses than in the Non-genotyping group, while Arg/Cys patients in the Genotyping group began at lower ones than those in the Non-genotyping group (*p* = 6.82E−6 and 1.78E−27, respectively). Furthermore, in the Genotyping group, more Arg/Arg patients received standard doses, while Arg/Cys patients predominantly received low or very low doses, with only 4.5% exceeding the standard doses (Fig. 1e). In both Non-genotyping and Genotyping groups, the maintenance dose was significantly lower for Arg/Cys than for Arg/Arg (*p* = 8.18E−6 and 6.84E−21, respectively).

### Frequency of AEs was significantly lower in Genotyping group

AEs within the first 2 years after thiopurine induction were compared between the Non-genotyping and Genotyping groups (Table 2). Overall, 38.1% of Non-genotyping and 27.1% of Genotyping patients experienced AEs, with these rates differing significantly (*p* = 7.31E−9). The discontinuation rate was 75.1% in the Non-genotyping group and 58.0% in the Genotyping group (*p* = 5.82E−7). In addition, 19.4% in the Non-genotyping group needed additional drug treatment and/or hospitalization, while the corresponding rate in the Genotyping group was 9.3% (*p* = 6.97E−5). Leukopenia and alopecia were less common in the Genotyping group, with severe cases being rare. Conversely, other major AEs such as nausea and liver injury were not associated with prior genotyping.

**Table 2:** Comparison of first adverse events within 2 years of thiopurine induction

	Non-genotyping (*n* = 1010)	Genotyping (*n* = 1512)	*p*-Value
Action for thiopurine at AE			
Reduction	96 (24.9)	172 (42.0)	5.82E−07^*^
Discontinuation	289 (75.1)	238 (58.0)	
Action for AE, *n* (%)			
Reduction or discontinuation only	301 (79.0)	358 (87.5)	2.56E−04^*^
Additional treatment	20 (5.2)	14 (3.4)	
Hospitalization	54 (14.2)	24 (5.9)	
Other	6 (1.6)	13 (3.2)	
Thiopurine drug at AE, *n* (%)			
AZA	289 (75.9)	330 (80.5)	1.36E−01
6MP	92 (24.1)	80 (19.5)	
Thiopurine dose at AE, median (IQR)	50.0 (25.0, 50.0)	50.0 (25.0, 50.0)	9.63E−01
Any of all AEs, *n* (%)	385 (38.1)	410 (27.1)	7.31E−09^*^
Leukopenia (grade), *n* (%)			
Grade 1	24 (2.4)	30 (2.0)	
Grade 2	92 (9.2)	46 (3.0)	
Grade 3	41 (4.1)	9 (0.6)	
Grade 4	17 (1.7)	1 (0.1)	
Grade unknown	6 (0.6)	–	–
All leukopenia, *n* (%)			
Any grade	180 (17.9)	86 (5.7)	2.68E−22^*^
Severe leukopenia, *n* (%)			
Grade 3 or 4	58 (5.8)	10 (0.7)	1.97E−14^*^
Alopecia, *n* (%)			
All	66 (6.6)	41 (2.7)	3.64E−06^*^
Mild (subjective)	35 (3.6)	39 (2.6)	1.76E−01
Severe	31 (3.1)	2 (0.1)	4.96E−10^*^
Nausea, *n* (%)	73 (7.3)	120 (7.9)	6.17E−01
Liver injury, *n* (%)	51 (5.1)	85 (5.6)	6.28E−01
Fever, *n* (%)	31 (3.1)	31 (2.1)	1.23E−01
Hyperamylasemia, *n* (%)	16 (1.6)	16 (1.1)	3.28E−01
Pancreatitis, *n* (%)	13 (1.3)	21 (1.4)	9.73E−01
Fatigue, *n* (%)	14 (1.4)	22 (1.5)	1.00E+00
Headache, *n* (%)	11 (1.1)	15 (1.0)	9.70E−01
Abdominal pain, *n* (%)	2 (0.2)	13 (0.9)	6.40E−02
Skin lesion, *n* (%)	4 (0.4)	10 (0.7)	5.46E−01
Joint and muscle pain, *n* (%)	4 (0.4)	8 (0.5)	7.72E−01
Infection, *n* (%)	5 (0.5)	5 (0.3)	5.34E−01
Stomatitis, *n* (%)	4 (0.4)	6 (0.4)	1.00E+00
Diarrhea, *n* (%)	4 (0.4)	4 (0.3)	7.21E−01
Anemia, *n* (%)	4 (0.4)	4 (0.3)	7.21E−01
Pneumonia, *n* (%)	6 (0.6)	0 (0)	4.07E−03^*^
Vertigo, *n* (%)	3 (0.3)	2 (0.1)	3.95E−01
Thrombocytopenia, *n* (%)	4 (0.4)	0 (0)	2.56E−02^*^
Malignant tumor, *n* (%)	1 (0.1)	1 (0.1)	1.00E+00
Others, *n* (%)	6 (0.6)	11 (0.8)	8.94E−01

^*^p < 0.05

### Decreased incidence of AEs and increased treatment retention in Arg/Cys

**Fig. 2:**
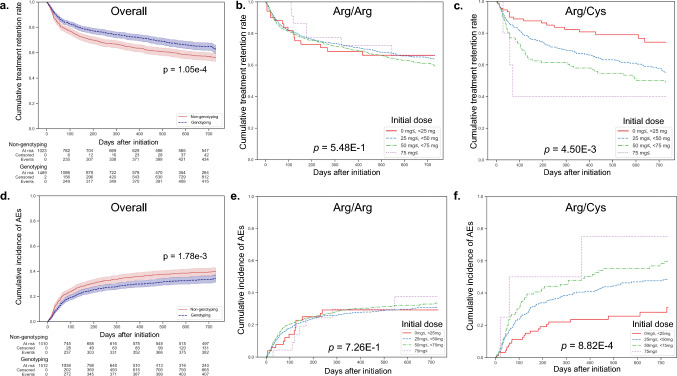
Comparison of cumulative treatment retention and adverse event incidence with and without NUDT15 Genotyping. Cumulative treatment retention rate (**a–c**) and cumulative incidence of AEs (**d–f**) were analyzed. The *x*-axis represents the number of days since the initiation of thiopurine treatment. **a, d** comparisons for the entire patient cohort for the Genotyping group (blue) and Non-genotyping group (red). **b, c, e, f** comparisons according to initial dose groups: very low dose (< 25 mg, red), low dose (25 mg to < 50 mg, blue), standard dose (50 mg to < 75 mg, green), and high dose (≥ 75 mg, purple). **b, e** Arg/Arg patients and **c, f** Arg/Cys patients. *p*-values are from the log-rank method. AE, adverse event

Treatment retention rates were significantly higher in the Genotyping group (72.4% and 62.6% at 1 and 2 years) than in the Non-genotyping group (64.3% and 56.2%, *p* = 1.05E−4; Fig. 2a). Arg/Arg patients showed no difference in retention with or without genotyping (*p* = 2.81E−1), while Arg/Cys patients experienced higher retention rates in the Genotyping group (*p* = 2.22E−7) (Supplementary Figure S1a, b). Similar results were observed for AE incidence (Fig. 2d, Supplementary Figure S1c, d).

### Age was the only risk factor for treatment discontinuation in Genotyping group

Comparison of genotypes revealed lower treatment retention rates and higher AE incidence for both Arg/Cys and Cys/Cys in the Non-genotyping group (Supplementary Figure S2a, c). In the Genotyping group, there was no difference in AE incidence between Arg/Arg and Arg/Cys (*p* = 9.77E−1), and treatment retention rates were slightly higher for Arg/Cys, although the difference was not statistically significant (*p* = 5.60E−2; Supplementary Figure S2b, d). Multivariate analysis showed that, in the Genotyping group, risk factors for AEs included sex, age, concomitant XO inhibitor use, and the absence of 5-ASA use (Supplementary Table S3). Only age at thiopurine therapy initiation emerged as a risk factor for treatment discontinuation. The genotypes of NUDT15 (Arg/Cys, Cys/Cys) showed no correlation with AEs or treatment discontinuation in the Genotyping group.

### Higher dose at thiopurine initiation is a risk factor for AEs in Arg/Cys but not in Arg/Arg

The risk of AEs was examined concerning the initial dose of thiopurines. For Arg/Arg, no significant differences in AE incidence based on initial dose were observed (*p* = 7.26E−1; Fig. 2e). Conversely, for Arg/Cys, dose-dependent differences were noted, with AE incidence being dose dependent (*p* = 8.82E−4; Fig. 2f). Similar patterns were seen in treatment retention rates (Fig. 2b, 2c).

**Fig. 3:**
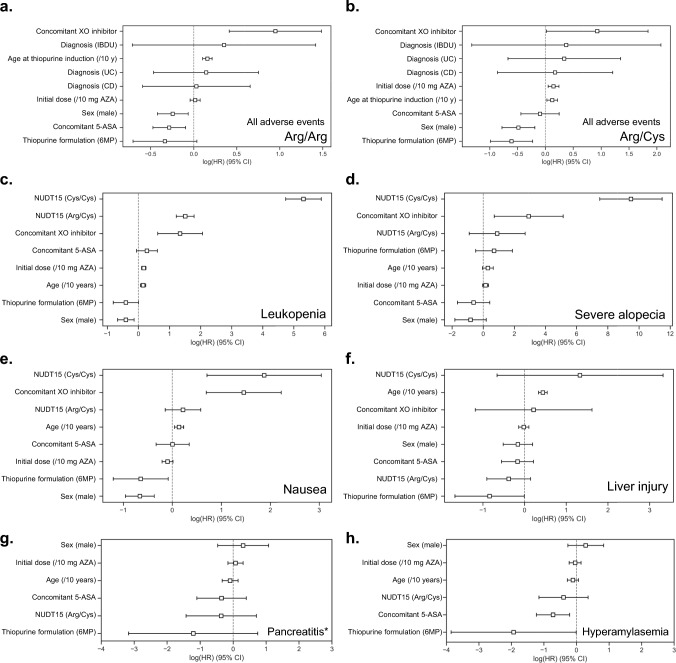
Risk factors for adverse events. The Cox proportional hazards model was used to analyze risk factors for the incidence of AEs stratified by genotype: **a** Arg/Arg and **b** Arg/Cys and major AEs, including leukopenia (**c**), severe alopecia (**d**), nausea (**e**), liver injury (**f**), pancreatitis confirmed by imaging (**g**) and hyperamylasemia (**h**), following thiopurine induction. White boxes indicate hazard ratios for each factor, with lines indicating their 95% CI. HR, hazard ratio; CI, confidence interval; IBD, inflammatory bowel disease; AZA, azathioprine; CD, Crohn’s disease; UC, ulcerative colitis; BD, intestinal Behçet’s disease; IBDU, inflammatory bowel disease, unclassified; AZA, azathioprine; 6MP, 6-mercaptopurine; 5-ASA. 5-aminosalicylic acid; XO, xanthine oxidase

Multivariate analysis confirmed these findings (Table 3, Fig. 3a, b, Supplementary Figure S3). In Arg/Cys, the initial dose of thiopurines was identified as a risk factor for AEs and treatment discontinuation. Concomitant use of a 5-ASA formulation was protective against AEs in those with the Arg/Arg genotype and against treatment discontinuation in those with Arg/Cys. Regardless of genotype, starting thiopurines at an older age and AZA (as opposed to 6MP) were risk factors for AEs and treatment discontinuation.

**Table 3:** Risk factors for incidence of AEs and treatment discontinuation by NUDT15 genotype

	Incidence of AEs				Treatment discontinuation			
	Arg/Arg		Arg/Cys		Arg/Arg		Arg/Cys	
	*p*-Value	HR (95% CI)	*p*-Value	HR (95% CI)	*p*-Value	HR (95% CI)	*p*-Value	HR (95% CI)
Sex								
Male	7.42E−03^*^	0.78 (0.66–0.94)	1.29E−03^*^	0.62 (0.46–0.83)	1.64E−01	0.89 (0.75–1.05)	1.20E−01	0.78 (0.57–1.07)
Female		(reference)		(reference)		(reference)		(reference)
Age at thiopurine induction								
(/10 years)	2.06E−08^*^	1.17 (1.11–1.24)	1.19E−02^*^	1.13 (1.03–1.24)	4.85E−03^*^	1.08 (1.02–1.14)	8.63E−03^*^	1.14 (1.03–1.26)
Diagnosis								
CD	9.22E−01	1.03 (0.55–1.93)	7.43E−01	1.19 (0.42–3.34)	4.95E−01	0.81 (0.45–1.47)	9.98E−01	1.00 (0.35–2.83)
UC	6.44E−01	1.15 (0.63–2.12)	5.13E−01	1.40 (0.51–3.85)	7.17E−01	1.11 (0.62–1.99)	7.11E−01	1.21 (0.44–3.34)
IBDU	5.17E−01	1.42 (0.49–4.13)	6.66E−01	1.45 (0.27–7.95)	1.74E−01	1.87 (0.76–4.61)	7.13E−01	1.38 (0.25–7.53)
BD		(reference)		(reference)		(reference)		(reference)
Thiopurine formulation								
6MP	7.72E−02	0.72 (0.49–1.04)	1.70E−03^*^	0.54 (0.37–0.80)	4.35E−02^*^	0.70 (0.50–0.99)	3.42E−03^*^	0.54 (0.36–0.82)
AZA		(reference)		(reference)		(reference)		(reference)
Initial dose (AZA)								
(/10 mg/day)	5.98E−01	1.02 (0.96–1.08)	2.98E−03^*^	1.16 (1.05–1.28)	9.40E−01	1.00 (0.95–1.06)	6.15E−03^*^	1.15 (1.04–1.27)
Concomitant 5-ASA								
Yes	3.16E−03^*^	0.75 (0.62–0.91)	5.81E−01	0.91 (0.64–1.28)	5.94E−02	0.84 (0.70–1.01)	4.22E−02^*^	0.70 (0.50–0.99)
Concomitant XO inhibitor								
Yes	5.33E−04^*^	2.58 (1.51–4.42)	4.54E−02^*^	2.53 (1.02–6.31)	5.59E−02	1.75 (0.99–3.11)	7.54E−02	2.27 (0.92–5.62)

*AE* adverse event, *HR* hazard ratio, *CI* confidence interval, *CD* Crohn’s disease, *UC* ulcerative colitis, *IBDU* inflammatory bowel disease, unclassified, *AZA* azathioprine, *6MP* 6-mercaptopurine, *5-ASA* 5-aminosalicylic acid, *XO* xanthine oxidase

*p < 0.05

### Higher maintenance doses are associated with higher treatment retention in Arg/Arg

No significant difference in overall treatment retention rates was observed for Arg/Arg or Arg/Cys at the maintenance dose up to 5 years (Supplementary Figure S4). However, upon comparing between groups individually, higher AZA doses (≥ 75 mg) had significantly higher treatment retention rates than lower and standard doses (≥ 25 mg to < 50 mg, and ≥ 50 mg to < 75 mg) in Arg/Arg (*p* = 4.98E−2 and 2.33E−3, respectively). Multivariate analysis confirmed a lower treatment discontinuation rate with a higher maintenance dose in Arg/Arg [*p* = 2.20E−3, hazard ratio [HR] (95% Confidence Interval [CI]) = 0.93 (0.89–0.97)], but no factors were associated with long-term treatment retention in Arg/Cys (Supplementary Table S4). Comparison of treatment retention rates by genotype with and without prior genotyping revealed a significantly lower long-term retention rate for Arg/Cys in the Non-genotyping group, while the Genotyping group exhibited no difference in retention rates (*p* = 1.83E−2 and 9.02E−1, respectively) (Supplementary Figure S5).

### Nausea and liver injury appear earlier than leukopenia

Analysis of the time from thiopurine treatment initiation to AE onset showed that leukopenia had a median onset of 160.0 (49.0, 456.0) days and a mean of 442.7 ± 714.1 days. Meanwhile, nausea and liver injury had median onsets of 61.0 (28.0, 124.5) and 50.5 (30.2, 97.0) days, with means of 222.1 ± 542.0 and 236.4 ± 686.3 days, respectively, occurring earlier than leukopenia. Infection, thrombocytopenia, and malignancy had longer periods until onset, exceeding 900 days (Supplementary Figure S6, Supplementary Table S4).

### Nausea and liver injury are less common with 6-MP than with AZA, but are not dose related

Multivariate analysis identified several risk factors for common AEs (Fig. 3c–h, Supplementary Table S6). Leukopenia was associated with NUDT15 genotype, concomitant XO inhibitors, initial dose, AZA use, age, and female sex. Severe leukopenia was primarily linked to the NUDT15 genotype. Severe alopecia was associated with XO inhibitors and the NUDT15 genotype (Cys/Cys). Nausea risk factors included female sex, older age, AZA use, XO inhibitors, and the NUDT15 genotype (Cys/Cys). Risk factors for liver injury were older age and AZA use. Pancreatitis showed no significant correlation with the investigated variables, but hyperamylasemia risk factors included AZA use and the absence of 5-ASA use.

### Concomitant mesalamine formulations and dosage are associated with thiopurine-related AEs

Among patients taking mesalamine, decreased leukopenia risk but significantly increased pancreatitis risk were observed in pH-dependent mesalamine users compared with the rates in time-dependent users [*p* = 1.93E−2 and 3.10E−2, HR (95% CI) = 0.61 (0.41–0.92), 3.77 (1.13–12.6)] (Table 4). The incidences of nausea and liver injury were not associated with mesalamine type, but correlated with mesalamine dosage [*p* = 3.56E−2 and 2.57E−2, HR (95% CI) = 1.30 (1.02–1.66), 1.40 (1.04–1.87)].

**Table 4:** Risk factors for major thiopurine-related AEs in mesalamine users

	Leukopenia (All)		Nausea		Liver injury		Pancreatitis	
	*p*-Value	HR (95% CI)	*p*-Value	HR (95% CI)	*p*-Value	HR (95% CI)	*p*-Value	HR (95% CI)
Sex (male)								
Male	1.37E−02^*^	0.67 (0.49–0.92)	5.52E−04^*^	0.54 (0.38–0.77)	9.86E−01	1.00 (0.65–1.53)	4.81E−01	1.41 (0.54–3.66)
Female		(reference)		(reference)		(reference)		(reference)
Age								
(/10 years)	5.48E−05^*^	1.23 (1.11–1.35)	3.03E−02^*^	1.13 (1.01–1.26)	6.49E−14^*^	1.60 (1.42–1.81)	4.41E−01	0.89 (0.67–1.19)
Initial dose								
(/10 mg/day AZA)	2.47E−06^*^	1.24 (1.13–1.35)	1.40E−01	0.90 (0.79–1.03)	7.36E−01	0.98 (0.84–1.13)	5.49E−01	0.90 (0.65–1.26)
Thiopurine drug								
6MP	3.93E−02^*^	0.58 (0.35–0.97)	4.46E−02^*^	0.49 (0.24–0.98)	4.16E−01	0.70 (0.30–1.65)	4.55E−01	0.46 (0.06–3.51)
AZA		(reference)		(reference)		(reference)		(reference)
Initial dose (AZA)								
(/10 mg/day)	2.47E−06^*^	1.24 (1.13–1.35)	1.40E−01	0.90 (0.79–1.03)	7.36E−01	0.98 (0.84–1.13)	5.49E−01	0.90 (0.65–1.26)
Concomitant mesalamine								
pH-dependent	1.93E−02^*^	0.61 (0.41–0.92)	6.33E−01	0.90 (0.58–1.40)	8.63E−01	0.96 (0.58–1.58)	3.10E−02^*^	3.77 (1.13–12.6)
Time-dependent		(reference)		(reference)		(reference)		(reference)
Mesalamine dose								
Dose (g/day)	8.80E−01	0.98 (0.80–1.21)	3.56E−02^*^	1.30 (1.02–1.66)	2.57E−02^*^	1.40 (1.04–1.87)	9.36E−01	1.02 (0.56–1.88)
Concomitant XO inhibitor								
Yes	6.15E−02	2.65 (0.95–7.34)	5.36E−03^*^	4.22 (1.53–11.62)	3.91E−01	1.86 (0.45–7.74)	7.35E−01	0.03 (0–Inf)
No		(reference)		(reference)		(reference)		(reference)
NUDT15 codon 139								
Arg/Cys	7.70E−21^*^	4.53 (3.30–6.22)	1.36E−01	1.38 (0.90–2.09)	1.54E−01	0.63 (0.34–1.19)	6.13E−01	0.72 (0.20–2.56)
Cys/Cys	–	–	1.10E−02^*^	6.44 (1.53–27.09)	7.84E−01	0.05 (0–Inf)	7.83E−01	0.05 (0–Inf)
Arg/Arg		(reference)		(reference)		(reference)		(reference)

*AE* adverse event, *HR* hazard ratio, *CI* confidence interval, *AZA* azathioprine, *5-ASA* 5-aminosalicylic acid, *XO* xanthine oxidase

*p < 0.05

## Discussion

Thiopurines are still frequently used for treating IBD, and the number of prescriptions continues to increase in Japan. In this country, the NUDT15 gene test is the only pharmacogenomic test that can be used in IBD treatment and is covered by public health insurance [11]. To the best of our knowledge, this study is the first to examine how this test is used by physicians in the real world and to clarify the outcomes.

The timing and purpose of the genotyping test are crucial when a new test becomes available. In this study, approximately 75% of patients were tested when changing treatments, while 25% were screened without an immediate treatment change planned. The induction rate for thiopurines in screening cases was low, at approximately 10%, which may increase over time, but a longer time between testing and initiation of treatment increases the risk of unnecessary testing. Because this genotyping test is a one-time procedure based on germline-derived polymorphism, repeating it multiple times should be avoided. Ensuring proper storage and sharing of individual genetic test results is essential, as demonstrated in a recent study using Japanese claims data [11].

The genotyping test itself does not alter the treatment prognosis; it depends on the actions taken based on genotype. In Japan, guidelines suggest avoiding thiopurine preparations in Cys/Cys cases and using half the usual initial dose for Arg/Cys cases [8]. This study found that all Cys/Cys patients who underwent the genotyping test for medical treatment chose not to undergo thiopurine treatment. However, nine cases with the Arg/Cys genotype did not receive such treatment after the possession of this genotype was confirmed, mainly because of other treatment priorities. At least in these cases, Arg/Cys was considered a risk factor for thiopurine treatment. Arg/Cys is certainly a risk factor for leukopenia, and some reports suggest that it requires long-term attention [18, 19]. However, based on a larger dataset, this study showed no significant difference in treatment retention rates between Arg/Arg and Arg/Cys; Arg/Cys even tended to do slightly better. Therefore, Arg/Cys alone may not warrant treatment avoidance as long as dosage adjustments are made based on genotype, suggesting that Arg/Cys is not a risk factor for AEs or treatment discontinuation.

Optimizing thiopurine therapy hinges on tailoring dosages to individual genotypes. In Japan, the standard thiopurine dose for IBD is 50 mg/day of AZA, but initial doses often begin at lower levels because of concerns about AEs. In fact, more than half of the patients commence AZA at 25 mg/day or lower. Since the implementation of the genotyping test, over half of the Arg/Arg patients have been started on doses of 50 mg/day or higher, yet the incidence of AEs has not increased. Over 95% of Arg/Cys patients began with doses of 25 mg/day or lower, resulting in a significant reduction in side effects and an improved rate of treatment continuation. Hence, the NUDT15 genotyping test not only prevents severe AEs by avoiding Cys/Cys treatment but also optimizes therapy by adjusting initial doses, ultimately reducing overall AE frequency and enhancing treatment retention rates.

For Arg/Arg individuals, this study found that the initial dose of thiopurine had no significant impact on treatment discontinuation or AEs. Because nearly all Arg/Arg patients received initial doses of 75 mg or less, it is suggested that the risk of AEs remains consistent within this dose range. Conversely, in Arg/Cys patients, a dose-dependent risk was observed. Starting with less than 25 mg of AZA yielded a similar retention rate and incidence of side effects as in Arg/Arg patients. While this study could not directly assess treatment efficacy, the continuation rate may reflect treatment effectiveness to some extent. Moreover, reports have been published suggesting that a small dose of 6-MP in Arg/Cys cases is as effective as the standard dose in Arg/Arg cases [20]. Considering these findings, it appears preferable to initiate treatment for Arg/Cys patients with less than 25 mg, specifically 6-MP at 10 mg/day or AZA at 12.5 mg/day, in terms of both safety and dosage efficacy.

For maintenance therapy, in Arg/Cys, there was no change in the risk of AEs or treatment discontinuation based on dosage, suggesting that low-dose thiopurine is sufficient. However, in Arg/Arg, a significantly higher treatment retention rate was observed with high doses (≥ 75 mg/day) of AZA, implying better maintenance effects with at least 75 mg/day of AZA.

Nausea, liver injury, and pancreatitis rates remained unchanged despite NUDT15 genotype-based dose adjustments. Nausea correlated with thiopurine formulation, showing a lower incidence with 6-MP than with AZA. XO inhibitor use and Cys/Cys genotype were also risk factors for nausea, while Arg/Cys was not. These associations may reflect secondary symptoms linked to serious side effects, rather than the typical thiopurine-induced nausea. Pancreatitis and liver injury were unrelated to thiopurine dose. Pancreatitis has genetic links in those of European ancestry, but no such risk factors have been identified in Japanese patients [8, 21, 22].

5-ASA medications, often used in IBD, appear to protect against thiopurine-induced AEs in Arg/Arg. However, they can also amplify the effects of thiopurines and pose risks for dose-dependent AEs because of their impact on thiopurine S-methyltransferase activity [23, 24]. The source of this inverse correlation remains unclear. Mesalamine, a commonly used 5-ASA, takes various forms, including time-dependent and pH-dependent formulations, affecting its delivery within the gastrointestinal tract. An analysis limited to mesalamine users revealed associations of mesalamine type and dosage with thiopurine-induced AEs. A pH-dependent formulation reduced the risk of leukopenia, possibly because of reduced mesalamine absorption in the small intestine [25]. The reason for fewer thiopurine-induced AEs occurring in Arg/Arg individuals using 5-ASA remains unclear. Very few Japanese IBD (especially UC) cases lack concurrent 5-ASA use [26, 27]. Thiopurine AEs might be indirectly linked to conditions unresponsive to or intolerant of 5-ASA, necessitating further study on the side effects of 5-ASA and thiopurines.

Based on the above, we propose the following treatment strategies of thiopurines for Japanese patients with IBD (Supplementary Table S7). For Cys/Cys, the avoidance of thiopurines is essential. In Arg/Arg cases, it is safe to initiate thiopurine treatment at 50 mg/day of AZA and then escalate to a maintenance dose of 75 mg/day or higher because the dose is not correlated with AEs. Those with the Arg/Cys genotype should start with a dose of 25 mg of AZA every other day or 10 mg/day of 6-MP. Dose adjustments can be made based on clinical responses, but there is generally no need to escalate to higher doses, with maintenance approximately 25 mg of AZA being the aim. When nausea or liver injury arises, switching from AZA to 6-MP may be considered. Irrespective of genotype, there is a need for vigilance about the possibility of AEs in the elderly, women, and those on concurrent XO inhibitors. In UC patients using mesalamine, switching to a pH-dependent mesalamine formulation may help if leukopenia occurs. If nausea or hepatotoxicity appears, reducing the mesalamine dosage instead of thiopurine might be more effective.

Despite the many findings of this study, there are several limitations. This work focused on treatment retention and AEs, with thiopurine treatment efficacy not being directly assessed. A lack of data on IBD activity and other therapies might influence AE assessments. In addition, given the retrospective and observational nature of this study, genotype-based treatment strategies varied. Moreover, while other polymorphisms in NUDT15 have been linked to AEs, the test kit approved for use in Japan solely genotypes codon 139, leaving the association with these other polymorphisms unexplored [6, 28, 29]. Furthermore, this study does not delve into the rare genotypes containing the His allele, despite severe leukopenia having been documented in His/His patients [30]. Despite these limitations, this study is to the best of our knowledge the first to depict real-world clinical NUDT15 gene testing in Japan, where it was applied for the first time globally. The significance of this work lies in showing how NUDT15 codon 139 genotyping reduces thiopurine AEs and enhances treatment retention rates. It also provides essential evidence for genotype-based prescribing, paving the way for personalized treatment strategies. Recently, concerns have been raised that this NUDT15 genotype may be associated with a fetal risk from maternal thiopurine administration during pregnancy, and this genetic test may be useful for assuaging such concerns, and in various other situations [31, 32]. Future research should explore long-term treatment efficacy, the test’s utility in various diseases, and its role in determining treatment strategies based on test results.

The greatest limitation of this study is the assessment of clinical efficacy, which needs to be conducted for each disease and the specific use of thiopurine. Given the wide range of available treatment options, studying thiopurine’s effectiveness presents difficulties. However, within the realm of IBD, the potential for evaluating thiopurine’s efficacy in managing steroid-dependent ulcerative colitis and in mitigating postoperative recurrence in Crohn’s disease exists, particularly through the retrospective examination of genotypes. Additionally, with the commercial availability of the NUDT15 gene test, it is advisable for future clinical studies to include the NUDT15 genotype data, potentially leading to the generation of new insights.

In conclusion, this study affirmed the effectiveness of NUDT15 codon 139 genotyping in lowering thiopurine-induced AEs and enhancing treatment retention among IBD patients. It also outlined personalized treatment strategies based on genotype, optimizing starting and maintenance doses while addressing major side effects of thiopurines and ways to manage them.

### Supplementary Information

Below is the link to the electronic supplementary material.

Supplementary file1 (PDF 1414 KB)

## Abbreviations

IBD: Inflammatory bowel disease
UC: Ulcerative colitis
CD: Crohn’s disease
BD: Intestinal Behçet’s disease
IBDU: Inflammatory bowel disease, unclassified
AZA: Azathioprine
6-MP: 6-Mercaptopurine
AE: Adverse event
WBC: White blood cell
