# Supplementary material for: Real-world NUDT15 genotyping and thiopurine treatment optimization in inflammatory bowel disease: a multicenter study
Source: J Gastroenterol. 2024 Apr 8;59(6):468–82. doi: 10.1007/s00535-024-02099-7 (PMC11128409; doi:10.1007/s00535-024-02099-7)

Supplementary Materials

Real-world NUDT15 genotyping and thiopurine treatment optimization in inflammatory bowel disease: A multicenter study

Tables

Supplementary Table S1. Patient characteristics

Supplementary Table S2. Initial and maintenance doses of thiopurines by genotype

Supplementary Table S3. Risk factors for AEs and treatment discontinuation with and without prior genotyping

Supplementary Table S4. Risk factors for treatment discontinuation in cases of long-term treatment by genotype of NUDT15

Supplementary Table S5. Days from the initiation of thiopurines to the onset of side effects

Supplementary Table S6. Risk factors for major AEs

Supplementary Table S7. Proposed thiopurine treatment strategies and summary of risk factors by NUDT15 genotype for Japanese patients with IBD

Figures

Supplementary Figure S1. Comparison of Cumulative Treatment Retention and Adverse Event Incidence with and without NUDT15 Genotyping

Supplementary Figure S2. Cumulative Treatment Retention and Adverse Event Incidence Stratified by NUDT15 Genotypes

Supplementary Figure S3. Risk Factors for Treatment Retention by NUDT15 Genotypes

Supplementary Figure S4. Cumulative Treatment Retention and Adverse Event Incidence by Maintenance Thiopurine Doses

Supplementary Figure S5. Cumulative Treatment Retention Rate with and without NUDT15 Genotyping at the Maintenance Dose

Supplementary Figure S6. Timing of Adverse Event Onset by Symptom

Supplementary Table S1. Patient characteristics

		All	Dataset A (Genotyped for medical purposes)	Dataset B (Thiopurine users)
Number of subjects		4628	2300	2744
Age at NUDT15 genotyping	Median (IQR)	37 (25–49)	35 (24–48)	36 (25–48)
Sex, n (%)	Male	2862 (61.8%)	1416 (61.6%)	1740 (63.4%)
	Female	1766 (38.2%)	884 (38.4%)	1004 (36.6%)
Disease, n (%)	UC	2816 (60.8%)	1447 (62.9%)	1653 (60.2%)
	CD	1683 (36.4%)	780 (33.9%)	1018 (37.1%)
	BD	85 (1.8%)	38 (1.7%)	48 (1.7%)
	IBDU	44 (1.0%)	35 (1.5%)	25 (0.9%)
NUDT15 genotyping prior to thiopurine use, n (%)	Non-genotyping	1214 (26.2%)	243 (10.6%)	1214 (44.2%)
	Genotyping	1530 (33.1%)	1173 (51.0%)	1530 (55.8%)
	unused	1884 (40.7%)	884 (38.4%)	-
NUDT15 codon 139, n (%)	Arg/Arg	3604 (77.9%)	1786 (77.7%)	2142 (78.1%)
	Arg/Cys	936 (20.2%)	476 (20.7%)	542 (19.8%)
	Cys/Cys	74 (1.6%)	28 (1.2%)	48 (1.7%)
	Arg/His	13 (0.3%)	10 (0.4%)	11 (0.4%)
	Cys/His	1 (0.0%)	0 (0%)	1 (0.0%)

IQR, interquartile range; UC, ulcerative colitis; CD, Crohn’s disease; BD, intestinal Behçet’s disease; IBDU, inflammatory bowel disease, unclassified

Supplementary Table S2. Initial and maintenance doses of thiopurines by genotype

Codon 139		Arg/Arg	Arg/Cys	Arg/His	Cys/Cys	Cys/His	p-value**
**Initial dose (mg/day)***							
Non-genotyping	n	744	224	2	40	1	
	Median (range)	25 (4.16–150)	25 (6.86–104)	25 (25–25)	25 (20–100)	25 (25–25)	7.58E–01
	Mean ± SD	36.7 ± 16.3	36.7 ± 16.6	25.0 ± 0.0	39.8 ± 21.2	25.0 ± 0.0	
Genotyping	n	1227	289	9	0	0	
	Median (range)	50 (10.4–150)	25 (6.24–50)	25 (20.8–50)	-	-	3.68E–68
	Mean ± SD	39.0 ± 14.0	24.5 ± 7.02	32.9 ± 12.9	-	-	
p-value*	Non-genotyping vs. Genotyping	6.82E–06	1.78E–27	-	-	-	
**Maintenance dose (mg/day)***							
Non-genotyping	n	554	137	1	0	0	
	Median (range)	50 (4.16–208)	50 (6.86–208)	50 (50–50)	-	-	8.18E–06
	Mean ± SD	60.2 ± 30.7	48.5 ± 28.4	50.0 ± NA	-	-	
Genotyping	n	718	181	5	0	0	
	Median (range)	50 (4.8–200)	41.6 (3.57–104)	50 (25–50)	-	-	6.84E–21
	Mean ± SD	62.8 ± 29.0	42.0 ± 21.7	45.0 ± 11.2	-	-	
p-value**	Non-genotyping vs. Genotyping	4.56E–02	3.63E–02	-	-	-	

*Equivalent to azathioprine, **Kruskal–Wallis test, NA, not available

Supplementary Table S3. Risk factors for AEs and treatment discontinuation with and without prior genotyping

		Incidence of AEs				Treatment discontinuation			
		Non-genotyping		Genotyping		Non-genotyping		Genotyping	
		**p-value**	**HR (95% CI)**	**p-value**	**HR (95% CI)**	**p-value**	**HR (95% CI)**	**p-value**	**HR (95% CI)**
**Sex**	Male	2.30E–02	0.77 (0.61–0.96)	1.26E–03	0.72 (0.59–0.88)	1.37E–01	0.85 (0.69–1.05)	1.26E–01	0.86 (0.70–1.04)
	Female		(reference)		(reference)		(reference)		(reference)
**Age at thiopurine induction**	(/10 years)	6.07E–04	1.14 (1.06–1.23)	1.66E–06	1.16 (1.09–1.23)	1.56E–01	1.05 (0.98–1.13)	4.72E–04	1.11 (1.05–1.18)
**Diagnosis**	CD	7.93E–01	0.90 (0.42–1.96)	7.74E–01	1.11 (0.53–2.32)	2.24E–01	0.67 (0.35–1.28)	7.88E–01	1.12 (0.49–2.59)
	UC	9.83E–01	0.99 (0.46–2.13)	4.13E–01	1.35 (0.66–2.75)	5.55E–01	0.82 (0.43–1.57)	2.14E–01	1.68 (0.74–3.79)
	IBDU	5.62E–01	1.44 (0.42–4.98)	9.51E–01	1.04 (0.27–3.99)	6.96E–01	1.26 (0.39–4.06)	1.39E–01	2.37 (0.75–7.47)
	BD		(reference)		(reference)		(reference)		(reference)
**Thiopurine formulation**	6MP	1.87E–02	0.64 (0.44–0.93)	1.89E–01	0.78 (0.54–1.13)	6.92E–03	0.61 (0.43–0.87)	1.41E–01	0.75 (0.51–1.10)
	AZA		(reference)		(reference)		(reference)		(reference)
**Initial dose (AZA)**	(/10 mg/day)	9.91E–01	1.00 (0.93–1.07)	6.82E–02	1.07 (0.99–1.15)	2.38E–01	1.04 (0.98–1.10)	6.02E–01	0.98 (0.91–1.06)
**Concomitant 5-ASA**	Yes	1.06E–01	0.80 (0.60–1.05)	9.76E–03	0.76 (0.62–0.94)	8.06E–03	0.71 (0.56–0.92)	1.21E–01	0.85 (0.69–1.04)
**Concomitant XO inhibitor**	Yes	2.18E–03	2.65 (1.42–4.93)	2.59E–02	2.13 (1.10–4.16)	2.45E–04	3.03 (1.68–5.49)	9.11E–01	1.05 (0.47–2.35)
**NUDT15 codon 139**	Arg/Cys	5.22E–12	2.29 (1.81–2.90)	1.69E–01	1.22 (0.92–1.63)	2.73E–04	1.54 (1.22–1.94)	1.93E–01	0.82 (0.61–1.10)
	Arg/His	4.24E–01	2.23 (0.31–16.0)	7.25E–01	1.23 (0.39–3.83)	3.35E–02	4.56 (1.13–18.5)	7.73E–01	1.18 (0.38–3.69)
	Cys/Cys	8.40E–39	20.0 (12.8–31.5)	-	-	2.84E–38	18.8 (12.0–29.2)	-	-
	Cys/His	9.89E–01	NA	-	-	9.89E–01	NA	-	-
	Arg/Arg		(reference)		(reference)		(reference)		(reference)

AE, adverse event; HR, hazard ratio; CI, confidence interval; CD, Crohn's disease; UC, ulcerative colitis; IBDU, inflammatory bowel disease, unclassified; AZA, azathioprine; 6MP, 6-mercaptopurine; 5-ASA, 5-aminosalicylic acid; XO, xanthine oxidase

Supplementary Table S4. Risk factors for treatment discontinuation in cases of long-term treatment by genotype of NUDT15

		Arg/Arg		Arg/Cys	
		**p-value**	**HR (95% CI)**	**p-value**	**HR (95% CI)**
**Sex**	Male	1.18E–01	1.23 (0.95–1.59)	5.33E–01	0.86 (0.54–1.37)
	Female		(reference)		(reference)
**Age at thiopurine induction**	(/10 years)	2.49E–01	0.95 (0.88–1.04)	5.58E–01	1.05 (0.90–1.22)
**Diagnosis**	CD	8.14E–01	1.15 (0.36–3.67)	9.95E–01	NA
	UC	3.44E–01	1.74 (0.55–5.49)	9.95E–01	NA
	IBDU	1.20E–01	4.18 (0.69–25.32)	9.95E–01	NA
	BD		(reference)		(reference)
**Thiopurine formulation**	6MP	7.45E–01	0.93 (0.61–1.42)	9.29E–01	1.02 (0.62–1.68)
	AZA		(reference)		(reference)
**Maintenance dose (AZA)**	(/10 mg/day)	2.20E–03*	0.93 (0.89–0.97)	4.23E–01	0.97 (0.88–1.05)
**Concomitant 5-ASA**	Yes	8.75E–01	1.02 (0.76–1.37)	9.89E–01	1.00 (0.57–1.73)
**Concomitant XO inhibitor**	Yes	9.89E–01	NA	3.16E–01	2.08 (0.50–8.73)

AE, adverse event; HR, hazard ratio; CI, confidence interval; CD, Crohn's disease; UC, ulcerative colitis; IBDU, inflammatory bowel disease, unclassified; AZA, azathioprine; 6MP, 6-mercaptopurine; 5-ASA, 5-aminosalicylic acid; XO, xanthine oxidase, *p<0.05

Supplementary Table S5. Days from the initiation of thiopurine to the onset of side effects

	n	Days to adverse events	
		Median (IQR)	Mean (SD)
**Leukopenia**	329	160.0 (49.0, 456.0)	442.7 (714.1)
**Nausea**	207	61.0 (28.0, 124.5)	222.1 (542.0)
**Liver injury**	146	50.5 (30.2, 97.0)	236.4 (686.3)
**Severe leukopenia**	78	48.5 (26.0, 158.5)	306.5 (703.6)
**Mild alopecia**	77	91.0 (54.0, 169.0)	169.9 (260.3)
**Fever**	65	30.0 (21.0, 91.0)	212.3 (754.3)
**Fatigue**	38	66.5 (31.2, 156.8)	230.3 (521.6)
**Hyperamylasemia**	36	29.5 (22.0, 80.0)	220.1 (646.1)
**Severe alopecia**	33	27.0 (23.0, 38.0)	43.5 (66.7)
**Pancreatitis**	32	27.0 (16.0, 50.8)	39.6 (39.6)
**Headache**	26	44.0 (28.0, 111.5)	77.4 (75.5)
**Skin lesion**	18	83.0 (13.2, 589.8)	366.6 (534.2)
**Infection**	16	84.0 (45.2, 1395.5)	941.1 (1539.6)
**Abdominal pain**	15	49.0 (24.5, 72.5)	69.1 (80.5)
**Joint and muscle pain**	12	25.0 (7.0, 106.8)	72.8 (107.9)
**Anemia**	11	189.0 (66.5, 771.5)	527.0 (621.8)
**Diarrhea**	9	14.0 (12.0, 30.0)	148.7 (360.8)
**Thrombocytopenia**	9	944.0 (511.0, 1274.0)	953.2 (644.3)
**Pneumonia**	8	211.5 (24.2, 812.0)	692.1 (1069.7)
**Vertigo**	5	22.0 (7.0, 25.0)	21.4 (17.3)
**Malignant tumor**	4	854.5 (438.8, 1591.0)	1175.2 (1176.2)
**Stomatitis**	3	105.0 (56.0, 214.0)	145.0 (161.8)

Supplementary Table S6. Risk factors for major AEs

		Leukopenia (all)		Severe leukopenia		Severe alopecia		Nausea	
		**p-value**	**HR (95% CI)**	**p-value**	**HR (95% CI)**	**p-value**	**HR (95% CI)**	**p-value**	**HR (95% CI)**
**Sex**	Male	2.85E–03*	0.67 (0.51–0.87)	5.60E–01	0.85 (0.50–1.46)	1.14E–01	0.44 (0.16–1.21)	1.00E–05*	0.52 (0.38–0.69)
	Female		(reference)		(reference)		(reference)		(reference)
**Age at induction**	(/10 years)	4.89E–04*	1.16 (1.07–1.27)	3.25E–01	1.09 (0.92–1.29)	9.13E–02	1.35 (0.95–1.91)	2.92E–03*	1.15 (1.05–1.26)
**Thiopurine formulation**	6MP	5.17E–02	0.67 (0.44–1.00)	9.64E–01	1.02 (0.46–2.25)	2.49E–01	2.00 (0.61–6.54)	2.44E–02*	0.52 (0.30–0.92)
	AZA		(reference)		(reference)		(reference)		(reference)
**Initial dose (AZA)**	(/10 mg/day)	2.32E–06*	1.19 (1.11–1.28)	3.32E–01	1.09 (0.92–1.29)	1.14E–01	1.16 (0.96–1.41)	9.01E–02	0.91 (0.81–1.02)
**Concomitant 5-ASA**		1.08E–01	1.32 (0.94–1.84)	8.41E–01	1.06 (0.58–1.96)	2.43E–01	0.54 (0.19–1.52)	9.76E–01	1.01 (0.72–1.41)
**Concomitant XO inhibitor**		2.53E–04*	3.82 (1.86–7.83)	5.05E–01	1.88 (0.29–12.0)	9.73E–03*	18.6 (2.03–170)	1.95E–04*	4.29 (1.99–9.23)
**NUDT15 codon 139**	Arg/Cys	3.56E–25*	4.50 (3.39–5.98)	8.44E–02	1.71 (0.93–3.13)	3.29E–01	2.45 (0.41–14.8)	2.38E–01	1.24 (0.87–1.78)
	Cys/Cys	6.28E–74*	203 (114–360)	1.54E–05*	>10000	1.55E–20*	>10000	1.67E–03*	6.51 (2.02–20.9)
	Arg/Arg		(reference)		(reference)		(reference)		(reference)

		Liver injury		Pancreatitis		Hyperamylasemia	
		**p-value**	**HR (95% CI)**	**p-value**	**HR (95% CI)**	**p-value**	**HR (95% CI)**
**Sex**	Male	3.72E–01	0.85 (0.60–1.21)	3.72E–01	1.35 (0.63–2.93)	3.12E–01	1.33 (0.77–2.30)
	Female		(reference)		(reference)		(reference)
**Age at induction**	(/10 years)	4.70E–17*	1.55 (1.40–1.72)	4.70E–17*	0.91 (0.72–1.16)	2.19E–01	0.90 (0.76–1.07)
**Thiopurine formulation**	6MP	4.78E–02*	0.43 (0.19–0.99)	4.78E–02*	0.30 (0.04–2.12)	4.89E–02*	0.14 (0.02–0.81)
	AZA		(reference)		(reference)		(reference)
**Initial dose (AZA)**	(/10 mg/day)	7.61E–01	0.98 (0.87–1.11)	7.61E–01	1.08 (0.85–1.36)	6.60E–01	0.96 (0.80–1.15)
**Concomitant 5-ASA**		3.90E–01	0.84 (0.58–1.24)	3.90E–01	0.70 (0.33–1.49)	5.66E–03*	0.49 (0.29–0.81)
**Concomitant XO inhibitor**		7.61E–01	1.24 (0.31–5.06)	7.61E–01	NA	5.97E–01	NA
**NUDT15 codon 139**	Arg/Cys	1.56E–01	0.68 (0.40–1.16)	1.56E–01	0.70 (0.24–2.03)	3.03E–01	0.67 (0.32–1.43)
	Cys/Cys	1.91E–01	3.78 (0.52–27.7)	1.91E–01	NA	6.83E–01	NA
	Arg/Arg		(reference)		(reference)		(reference)

AE, adverse event; HR, hazard ratio; CI, confidence interval; AZA, azathioprine; 5-ASA, 5-aminosalicylic acid; XO, xanthine oxidase

Supplementary Table S7. Proposed thiopurine treatment strategies and summary of risk factors by NUDT15 genotype for Japanese patients with IBD

<table>
<tr><th>NUDT15 codon 139</th><th>Arg/Arg</th><th>Arg/Cys</th><th>Cys/Cys</th></tr>
<tr><td>Initial dose*</td><td>50 mg/day</td><td>< 25 mg/day</td><td rowspan="2">Contraindication</td></tr>
<tr><td>Maintenance dose*</td><td>≥ 75 mg/day</td><td>Approximately 25 mg/day
(No need for higher doses)</td></tr>
<tr><td rowspan="2">Risk factors for AEs</td><td colspan="2">(Common)
● Sex (Female) – Leukopenia, Nausea
● Age (Older) – Leukopenia, Nausea, Liver injury
● Concomitant use of XO inhibitor – Leukopenia, Nausea</td><td rowspan="5"></td></tr>
<tr><td>(Arg/Arg only)
● No concomitant use of 5-ASA
*No change in risk with initial dose of thiopurine</td><td>(Arg/Cys only)
● Higher initiation dose – Leukopenia
● Formulation (AZA) – Leukopenia, Nausea, Liver injury</td></tr>
<tr><td rowspan="2">Risk factors for treatment retention</td><td colspan="2">(Common)
● Age (Older)
● Thiopurine formulation (AZA)</td></tr>
<tr><td>(Arg/Arg only)
● Low maintenance doses (<75 mg/day)
*No change in risk with initial dose of thiopurine</td><td>(Arg/Cys only)
● Higher initiation dose
● No concomitant use of 5-ASA</td></tr>
<tr><td>Risk factors for AEs in mesalamine users</td><td colspan="2">● Leukopenia: (Risk) Time-dependent mesalamine > pH-dependent mesalamine
● Pancreatitis: (Risk) pH-dependent mesalamine > Time-dependent mesalamine
● Nausea: (Risk) Higher dose of mesalamine > Lower dose of mesalamine
● Liver injury: (Risk) Higher dose of mesalamine > Lower dose of mesalamine</td></tr>
</table>

*Equivalent of azathioprine. AE, adverse event; XO, xanthine oxidase; 5-ASA, 5-aminosalicylic acid; AZA, azathioprine

Supplementary Figure Legends

Supplementary Figure S1. Comparison of Cumulative Treatment Retention and Adverse Event Incidence with and without NUDT15 Genotyping

Cumulative treatment retention rate (a,b) and cumulative incidence of AEs (c,d) were analyzed for the Genotyping group (blue) and Non-genotyping group (red). The x-axis represents the number of days since the initiation of thiopurine treatment. (a, b) focus on Arg/Arg patients, and (c, d) focus on Arg/Cys patients. AE, adverse event

Supplementary Figure S2. Cumulative Treatment Retention and Adverse Event Incidence Stratified by NUDT15 Genotypes

Cumulative treatment retention rate (a, b) and cumulative incidence of AEs (c, d) were analyzed for Arg/Arg (blue), Arg/Cys (green), and Cys/Cys (red). The x-axis represents the number of days since the initiation of thiopurine treatment. (a, c) are comparisons in the Non-genotyping group and (b, d) in the Genotyping group. p-values are from the log-rank method. AE, adverse event

Supplementary Figure S3. Risk Factors for Treatment Retention by NUDT15 Genotypes

The Cox proportional hazards model was used to analyze risk factors for treatment retention following thiopurine induction, stratified by genotype: (a) Arg/Arg and (b) Arg/Cys. White boxes indicate hazard ratios for each factor, with lines indicating their 95% CI. HR, hazard ratio; CI, confidence interval; IBD, inflammatory bowel disease; AZA, azathioprine; CD, Crohn's disease; UC, ulcerative colitis; BD, intestinal Behçet's disease; IBDU, inflammatory bowel disease, unclassified; AZA, azathioprine; 6MP, 6-mercaptopurine; 5-ASA. 5-aminosalicylic acid; XO, xanthine oxidase

Supplementary Figure S4. Cumulative Treatment Retention and Adverse Event Incidence by Maintenance Thiopurine Doses

Cumulative treatment retention rates were analyzed according to maintenance dose groups: very low dose (<25 mg, red), low dose (25 mg to <50 mg, blue), standard dose (50 mg to <75 mg, green), and high dose (≥75 mg, purple). (a) pertain to Arg/Arg patients and (b) to Arg/Cys patients. p-values are from the log-rank method. AE, adverse event

Supplementary Figure S5. Cumulative Treatment Retention Rate with and without NUDT15 Genotyping at the Maintenance Dose

Cumulative treatment retention rates at the maintenance dose up to 5 years with and without NUDT15 genotyping were compared. (a) represents the Non-genotyping group and (b) the Genotyping group. p-values were determined using the log-rank method.

Supplementary Figure S6. Timing of Adverse Event Onset by Symptom

For the timing of adverse drug reactions during the observation period for patients undergoing thiopurine treatment, circles indicate the mean number of days since the initiation of thiopurine and lines represent the standard deviation.

Supplementary Figure S1. Comparison of Cumulative Treatment Retention and Adverse Event Incidence with and without NUDT15 Genotyping

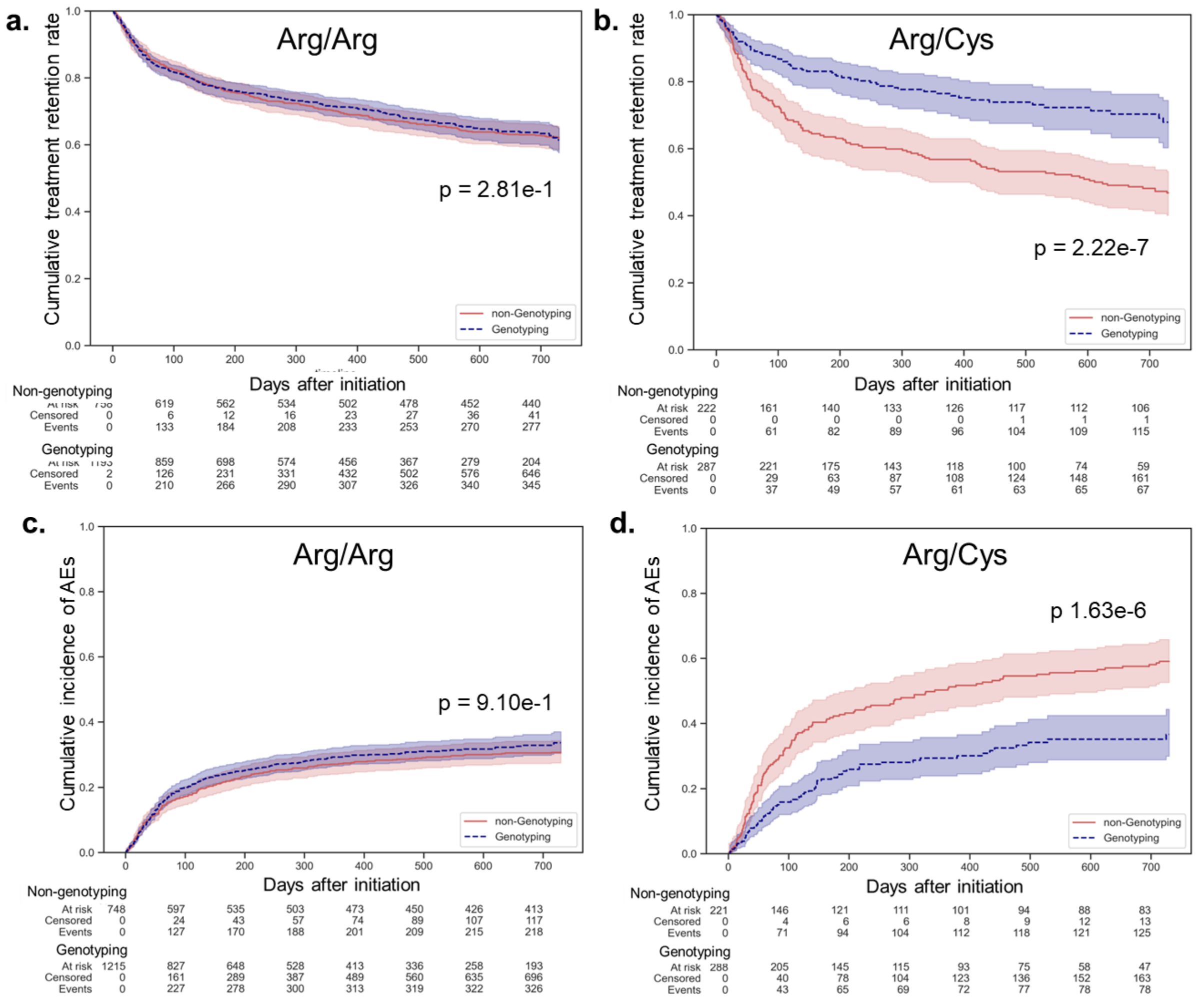

Supplementary Figure S2. Cumulative Treatment Retention and Adverse Event Incidence Stratified by NUDT15 Genotypes

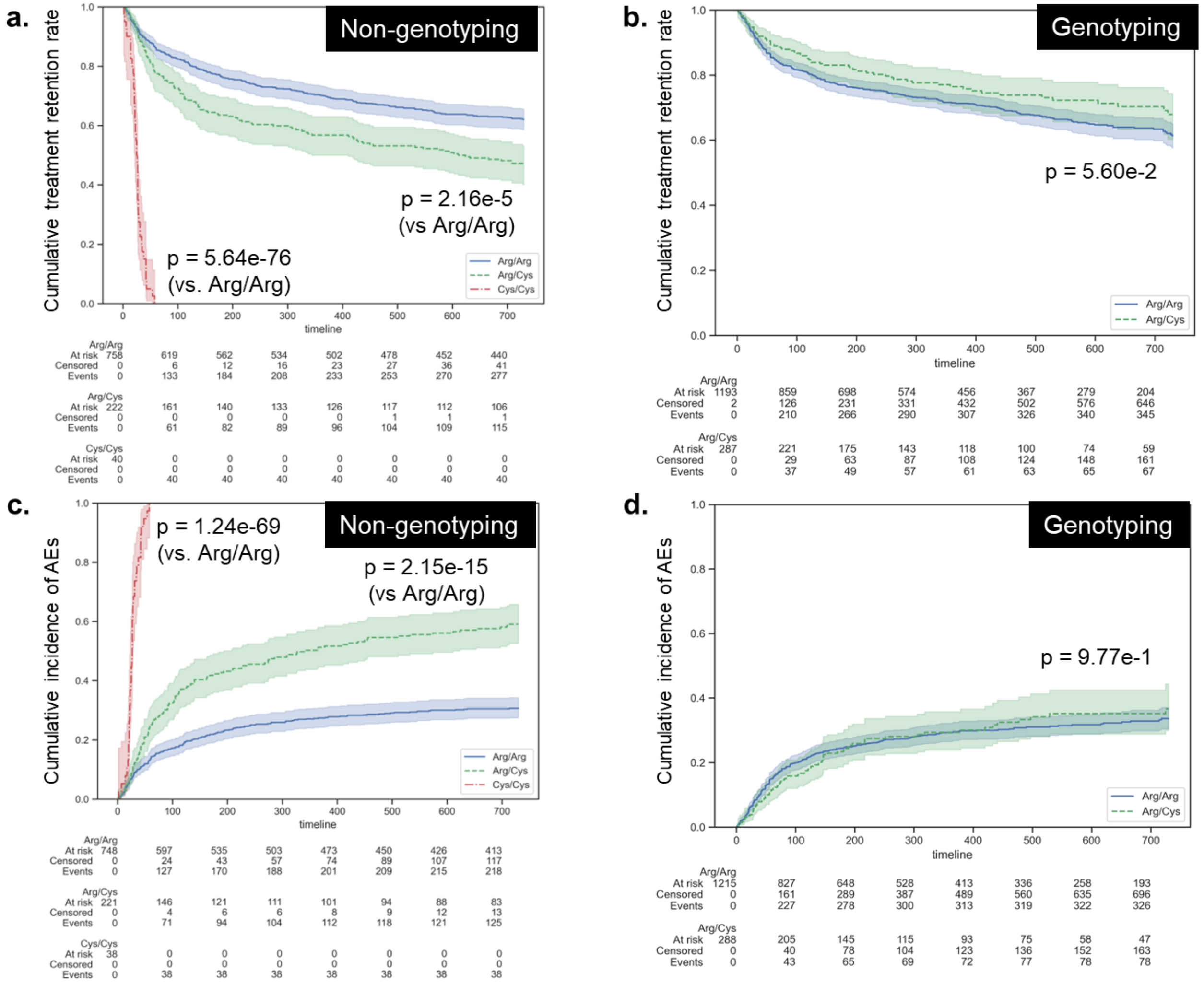

Supplementary Figure S3. Risk Factors for Treatment Retention by NUDT15 Genotypes

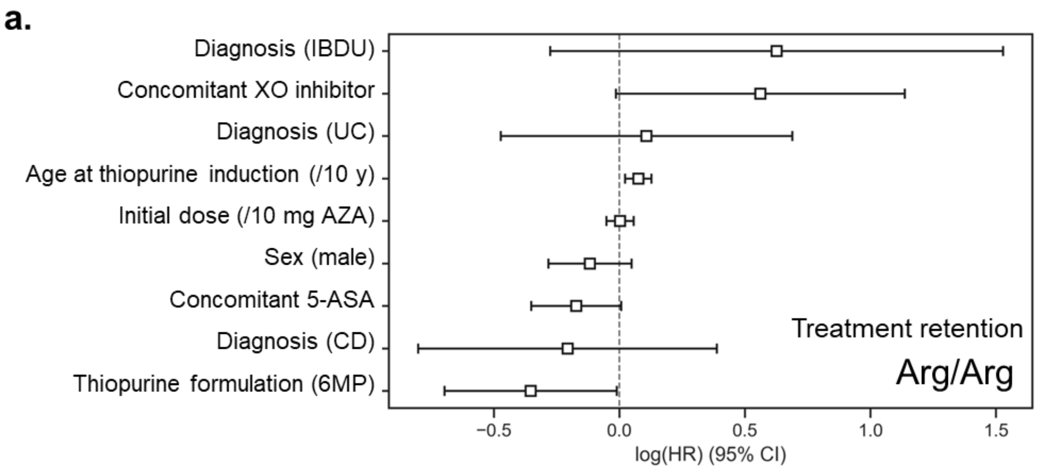

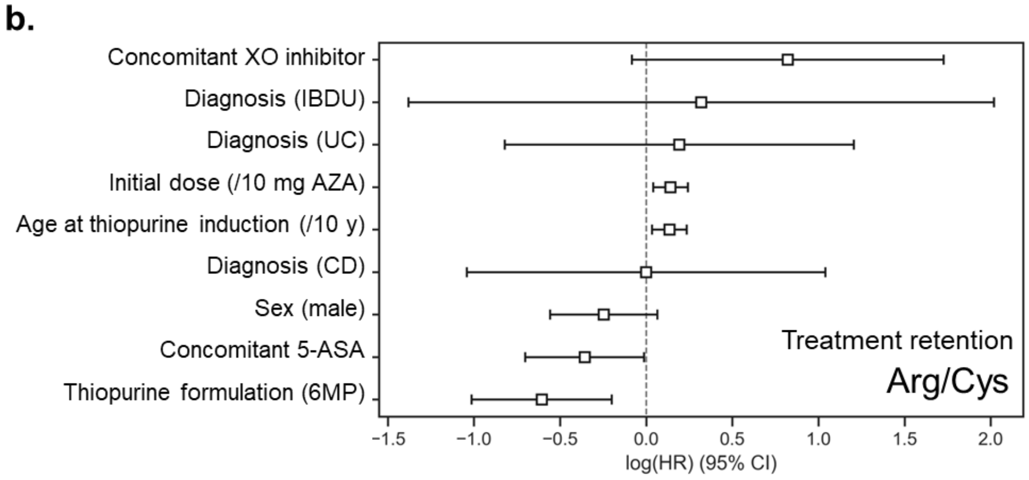

Supplementary Figure S4. Cumulative Treatment Retention and Adverse Event Incidence by Maintenance Thiopurine Doses

a.

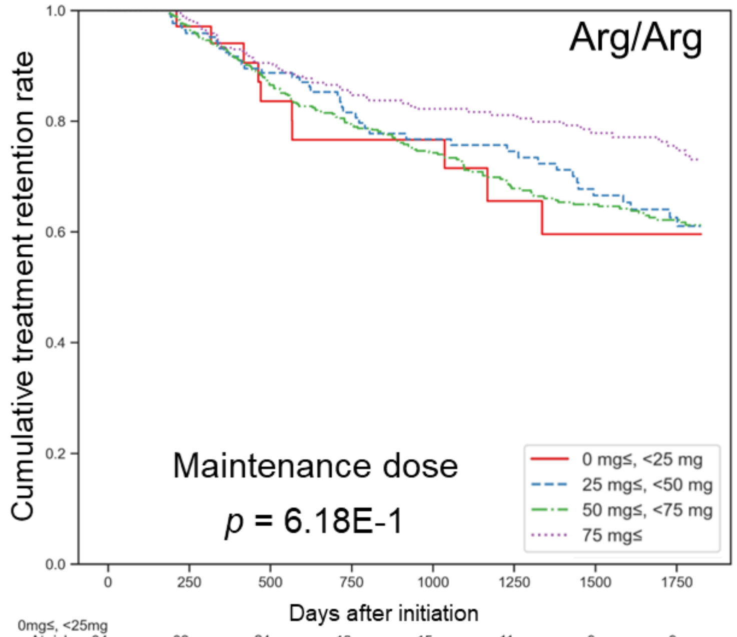

	0	250	500	750	1000	1250	1500	1750
**0mg≤, <25mg**								
At risk	34	32	24	18	15	11	9	9
Censored	0	1	5	9	12	14	15	15
Events	0	1	5	7	7	9	10	10
**25mg≤, <50mg**								
At risk	171	154	113	87	71	67	55	42
Censored	0	10	41	59	70	72	77	87
Events	0	7	17	25	30	32	39	42
**50mg≤, <75mg**								
At risk	656	605	424	305	241	196	170	142
Censored	0	33	155	242	289	314	332	353
Events	0	18	77	109	126	146	154	161
**75mg≤**								
At risk	411	380	276	182	156	137	110	91
Censored	0	24	101	181	201	218	240	255
Events	0	7	34	48	54	56	61	65

b.

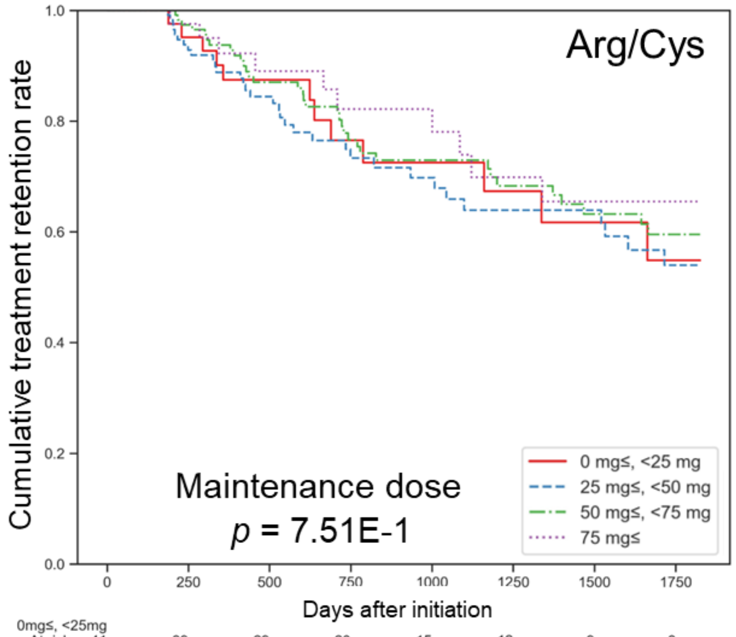

	0	250	500	750	1000	1250	1500	1750
**0mg≤, <25mg**								
At risk	41	39	29	20	15	12	9	8
Censored	0	0	7	13	17	19	21	21
Events	0	2	5	8	9	10	11	12
**25mg≤, <50mg**								
At risk	115	99	71	46	37	31	27	20
Censored	0	8	28	45	52	55	59	62
Events	0	8	16	24	26	29	29	33
**50mg≤, <75mg**								
At risk	121	110	86	63	53	44	36	30
Censored	0	8	21	35	42	48	53	57
Events	0	3	14	23	26	29	32	34
**75mg≤**								
At risk	41	39	28	23	20	16	14	9
Censored	0	1	9	12	15	16	17	22
Events	0	1	4	6	6	9	10	10

Supplementary Figure S5. Cumulative Treatment Retention Rate with and without NUDT15 Genotyping at the Maintenance Dose

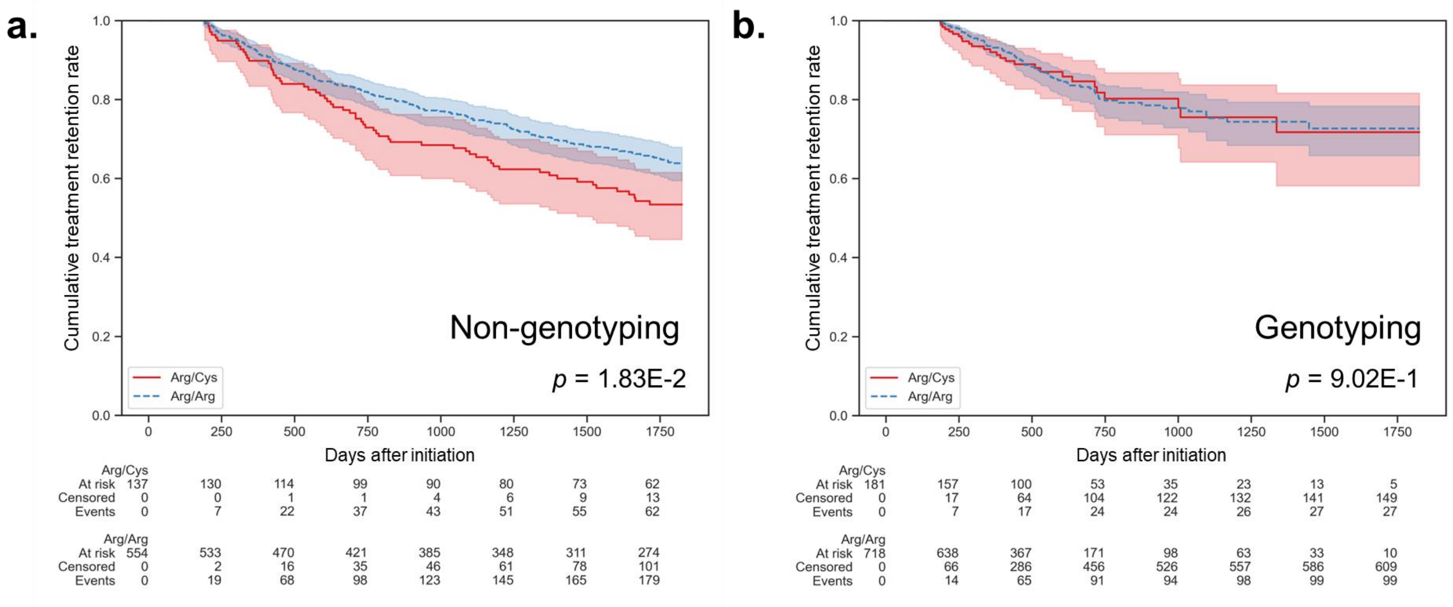

Supplementary Figure S6. Timing of Adverse Event Onset by Symptom

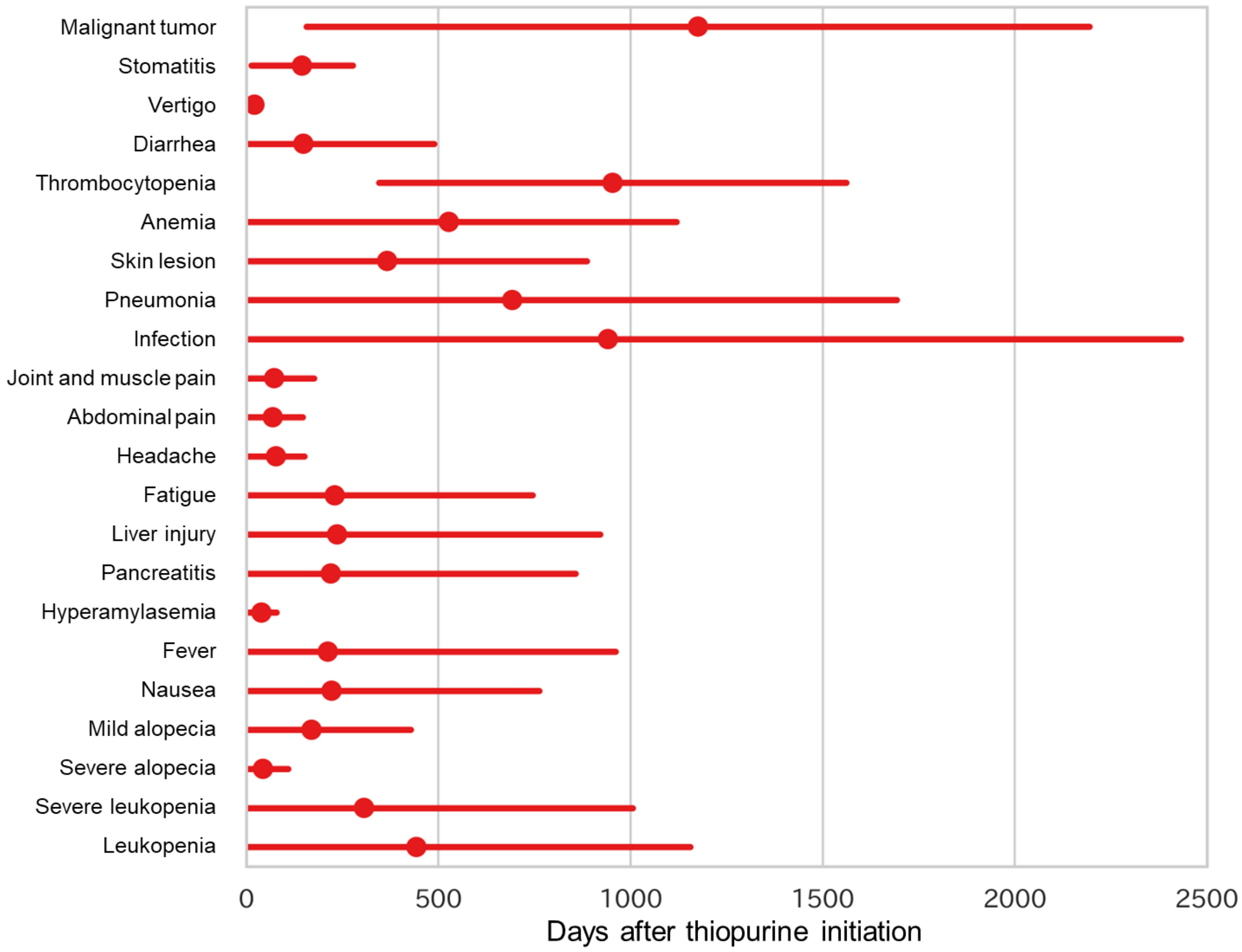

Supplement: Supplementary file 1 — Supplementary file1 (PDF 1414 KB) [file 535_2024_2099_MOESM1_ESM.pdf]
